# The antitumor mechanisms of aerobic exercise: A review of recent preclinical studies

**DOI:** 10.1002/cam4.4169

**Published:** 2021-08-13

**Authors:** Ningxin Jia, Yanan Zhou, Xiaosheng Dong, Meng Ding

**Affiliations:** ^1^ College of Physical Education Shandong Normal University Jinan China; ^2^ College of Physical Education Shandong University Jinan China

**Keywords:** aerobic exercise, antitumor, mechanism, signaling pathways

## Abstract

Aerobic exercise is an important non‐pharmacological means of antitumor intervention, but related mechanisms are poorly understood. In this review, previous studies are summarized from the aspects of tumor oxygenation, autophagy versus apoptosis, and organismal immunity. Current findings on the antitumor effects of aerobic exercise involve AMPK signaling, PI3K/Akt signaling, Th1/Th2 cytokine balance related to immunity, PD‐1/PD‐L1 immunosuppressive signaling, and related cytokine pathways. Several directions for further research are proposed, including whether newly discovered subgroups of cytokines influence the effects of aerobic exercise on tumors, tailoring corresponding exercise prescriptions based on the bidirectional effects of certain cytokines at different stages, identifying the potential effects of exercise time and intensity, and elucidating details of the unclear mechanisms. Through the discussion of the existing data, we hope to provide new ideas for the future research of exercise therapy.

## 1 BACKGROUND

Exercise is an important intervention for postoperative rehabilitation of oncology patients, and aerobic exercise is the main type of exercise intervention. The American College of Sports Medicine (ACSM) recommends that cancer survivors engage in at least 150–300 min/week of moderate intensity aerobic training.^1^ According to the cancer‐specific exercise guideline, an effective exercise prescription that most consistently addresses health‐related outcomes experienced due to a cancer diagnosis and cancer treatment includes moderate‐intensity aerobic training at least three times per week, for at least 30 min, for at least 8–12 weeks.^2^ As an adjunctive therapy for postoperative rehabilitation of cancer patients, aerobic exercise has the effect of enhancing conventional cancer treatments.^3^, ^4^ Available evidence suggests that aerobic exercise is associated with a reduction in tumor incidence, and has effects on affecting tumor physiology and controlling disease progression, which is associated with inhibition of tumor metastasis and recurrence.^5^, ^6^ Aerobic exercise interventions during the treatment of patients with cancer have a significant impact on their survival, and patients’ higher aerobic fitness leads to longer estimated tumor doubling times.^7^ A 2018 study found that 6 months of moderate intensity aerobic exercise significantly improves the quality of life of oncology survivors.^8^ Another study found that the degree of participation in exercise was inversely associated with postmenopausal breast cancer incidence.^9^ Aerobic exercise produces changes in physiological characteristics and induces systemic alterations under adaptations to long‐term training.^10^ Systemic effects of increased arterial pressure and cardiac output resulting from the rise in body temperature, as well as increased stroke volume and heart rate in response to acute exercise favor oxygen delivery, this effect can be further strengthened by the vascular remodeling induced by long‐term aerobic training.^11^ Preclinical studies have shown that aerobic exercise has been shown to induce vascular normalization in mouse models,^12^ which indicates that aerobic exercise may affect local physiological responses and vascular adaptations in the tumor microenvironment.

However, despite the existence of corresponding clinical evidence for the beneficial effects of aerobic exercise on tumors, the mechanisms by which aerobic exercise inhibits or retards tumorigenesis currently remain elusive. On the basis of previous studies, we speculate that aerobic exercise may inhibit tumor development and progression by improving vascular oxygenation, modulating autophagy versus apoptosis, and affecting immune function, and then teased out the regulatory mechanisms and signaling pathways implicated in these processes. Finally, providing some ideas that are worth exploring for further discussion.

## 2 MECHANISMS OF AEROBIC EXERCISE IN TUMOR HYPOXIA

### 2.1 Hypoxic tumor microenvironment

Due to the swift proliferation of tumors during their growth, the diffusion of oxygen into the surrounding capillaries is unable to meet the demands posed by the continuous propagation of tumor cells, causing tissue hypoxia as well as metabolic imbalance and resulting in the formation of a hypoxic tumor environment (TME).^13^ Mitochondrial metabolic reprogramming is a dynamic process in tumorigenesis and development and is essential for tumor growth.^14^ Mitochondrial metabolism supports tumor anabolism by providing key metabolites for macromolecule synthesis and generating oncometabolites to maintain the cancer phenotype.^15^ The flexibility of mitochondrial metabolism may meet different needs during various stages of tumorigenesis to metastasis.^14^ Mitochondrial dysfunction and aerobic glycolysis (Warburg effect) have been widely accepted as tumor markers. In TME, inhibition of mitochondrial metabolism is crucial to switch from oxidative phosphorylation to glycolysis.^16^ Hypoxia is a common feature of many solid tumors and plays an important role in tumor initiation, progression, and metastasis.^17^ HIF (hypoxia‐inducible factor) is a nuclear transcription factor that includes the members HIF‐1, HIF‐2, and HIF‐3.^18^ HIF‐1 is an important mediator in inducing aerobic glycolysis in tumor cells,^19^ HIF‐1 expression is increased under hypoxia and activates adaptive transcriptional responses through related signaling pathways, which involves the upregulation of survival factors including angiogenic factors, regulating energy metabolism within tumor cells to adapt the tumor to a hypoxic TME and promote tumor cell metastasis and invasion.^20^, ^21^, ^22^ Angiogenesis is a condition for tumor cells to grow and metastasize, and vascular endothelial growth factor (VEGF) can promote endothelial cell growth and increase vascular permeability.^23^, ^24^ Some studies have shown that under pathological conditions, HIF‐1 induces the activation of multiple drug‐resistance transporters by upregulating the protein expression of VEGF, leading to increased tumor malignancy.^23^ It has also been found that HIF‐1α is associated with angiogenesis in non‐small‐cell lung cancer (NSCLC), and the expression of HIF‐1α and VEGF are positively correlated and significantly higher in lung cancer tissues compared with normal tissues.^25^

### 2.2 Mechanisms of aerobic exercise on tumor perfusion and hypoxia

**FIGURE 1:**
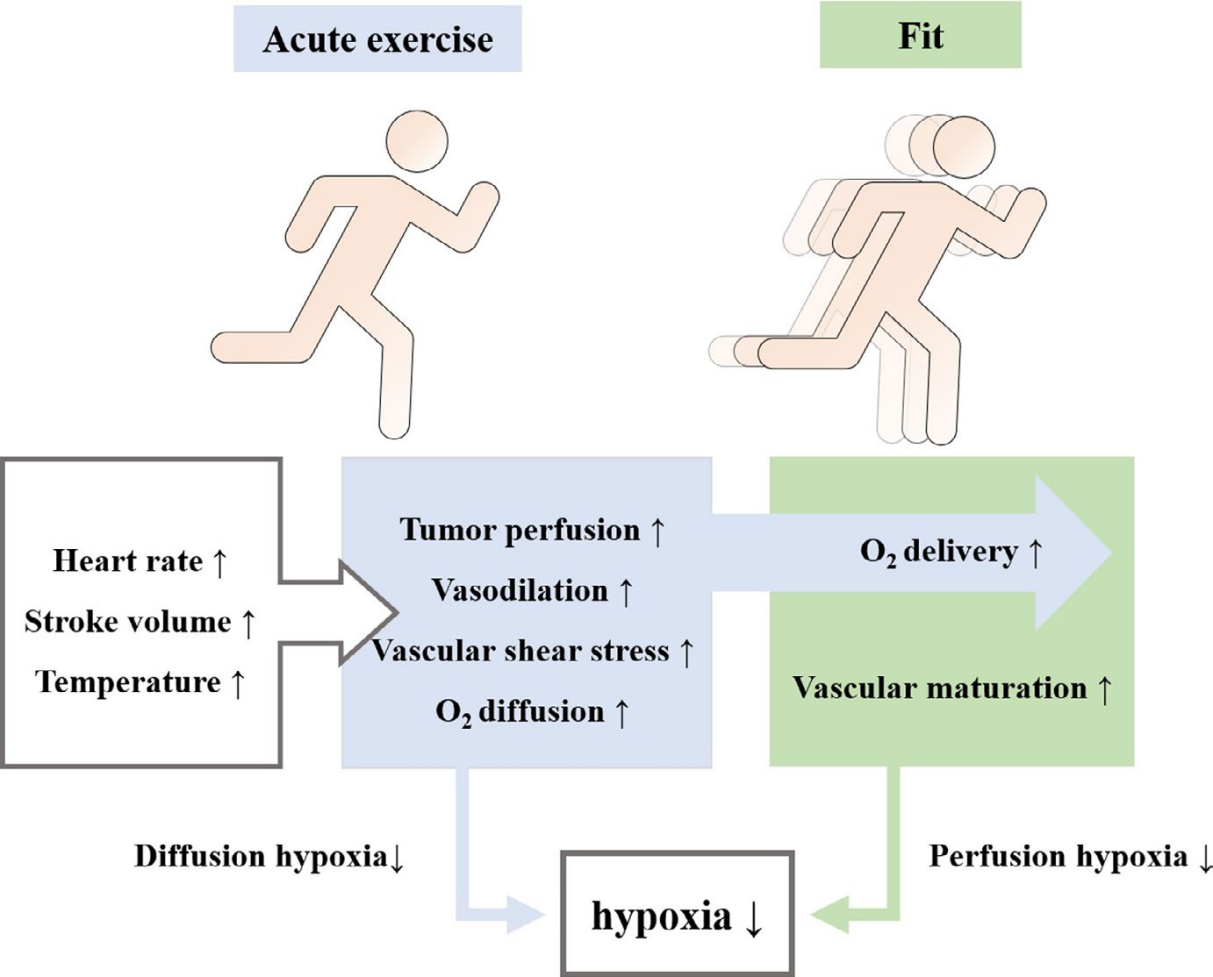
Mechanisms of aerobic exercise regulate tumor perfusion and hypoxia

Exercise has the effect of regulating tumor vasculature and oxygenation, and it may lead to the development of relatively normal, more mature, and less permeable vessels in dysfunctional tumor vasculature. This blunted vasoreactivity may allow for maintenance or increased tumor perfusion during exercise.^4^ Aerobic exercise induces robust changes in mitochondrial content and quality that are shown to enhance mitochondrial biogenesis, and are shown to delay or prevent impairments in mitochondrial functions.^26^ Peroxisome proliferator‐activated receptor γ coactivator‐1α (PGC‐1α) has been considered the master regulator of mitochondrial biogenesis.^27^ Acute aerobic exercise initiates a multitude of signals that activate PGC‐1α, with the most widely accepted signals being: calcium/calmodulin‐dependent protein kinase (CaMK), p38 mitogen‐activated protein kinase (p38 MAPK), and AMP‐activated protein kinase (AMPK) phosphorylation.^28^ By activating PGC‐1α redox signaling pathways, aerobic exercise stimulates mitochondrial biogenesis, which in turn reduces the risk of carcinogenesis.^28^ Reactive oxygen species (ROS) is important components of the tumor growth environment. ROS in tumor cells is mainly generated from mitochondria, and mitochondrial ROS activate the HIF signaling pathway to mediate tumor cell metabolism.^29^, ^30^, ^31^ The activity of HIF‐1 is regulated by hydroxylation. In hypoxia, the blockage of proline hydroxylation due to the inactivation of prolyl hydroxylase (PHD) and factor inhibiting HIF‐1 (FIH‐1) prevents the hydrolysis of HIF‐1α, inducing activation of HIF‐1 to affect the expression of VEGF, which drives tumor cell proliferation.^32^, ^33^, ^34^ Increased shear stress on endothelial cells during aerobic exercise is associated with tumor vascular remodeling, prompting greater blood flow to the tumor.^35^ Aerobic exercise alleviates hypoxia by improving tumor perfusion volume, serves to reduce the accumulation of mitochondrial ROS, which favors HIF‐1 inactivation.^36^, ^37^ The pressure exerted on the vessel wall under acute exercise conditions increases with increasing exercise intensity, promoting the development of functionally mature vessels. Acute exercise increases tumor blood flow and decreases tumor vascular resistance, increasing tumor perfusion pressure in a murine prostate cancer (PCa) model,^11^ the increase in oxygen diffusion distance enables the hypoxic tumor area with restricted diffusion to shrink. The adaptation of tumor vascular structure to long‐term exercise, can improve the oxygen delivery of peripheral tissue.^4^, ^11^, ^38^ Aerobic exercise exerts modulatory effects on tumor perfusion and hypoxia by enhancing acute and chronic oxygenation mechanisms in hypoxic tumor regions, which may help to delay tumor development^5^ (Figure 1).

### 2.3 Regulation of aerobic exercise on tumor brain metastasis

#### 2.3.1 Hypoxia and tumor brain metastasis

Metastasis is driven by inflammatory signals and infiltration of inflammatory cells into the primary tumor.^39^ The key steps in the initiation and growth of tumor metastasis depend on the ability of tumor cells to transverse the vascular membrane, enter the bloodstream, and penetrate endothelial cells at new sites.^40^ Tumor cells have a propensity to metastasize under hypoxic conditions, and hypoxia downregulates the expression of adhesion molecules and increases tumor cell motility.^41^ In addition, Rho is a redox sensitive small GTPase whose activation is associated with destabilization and increased permeability of the endothelial barrier during tumorigenesis. Tight junction proteins are major structural components that determine paracellular integrity of brain endothelium, which regulates endothelial cell permeability, and creates a barrier, through cell adhesion, that limits access to the cerebrospinal fluid environment.^42^, ^43^, ^44^ Studies have found that an increase in blood–brain barrier permeability is accompanied by a decrease in the expression of tight junction proteins,^44^, ^45^ whereas Rho kinase (ROCK), which is activated by RhoA GTPases, can induce phosphorylation of tight junction proteins, leading to their disruption and promoting the migration of monocytes through the blood–brain barrier.^46^ Tumor cells can interfere with the expression of tight junction proteins and extravasate and spread into the brain between disrupted tight junction proteins.^47^ Hypoxia causes the degradation of tight junction proteins, which further increases blood–brain barrier permeability.^44^ Redox‐sensitive small GTPases are involved in blood–brain barrier remodeling and barrier disruption, disrupts the structure of tight junction proteins, leading to tumor cell extravasation.^48^

#### 2.3.2 Mechanisms of vascular permeability to aerobic exercise

The redox stability of brain endothelial cells may protect the brain against tumor metastasis.^49^ Aerobic exercise maintains or increases the expression of tight junction proteins by regulating the activity of redox sensitive small GTPases, thereby affecting the oxidative status and redox sensitive signaling of cerebral microvessels to maintain blood–brain barrier integrity.^50^ An animal study found that at the early stage of tumor growth, the activation of RhoA in the microvessels of exercising mice was relatively low and the expression of tight junction proteins remained unchanged.^50^ In addition, aerobic exercise promotes VEGF expression in hippocampal tissue by inducing the proliferation of new vessels, and inducing peripheral tissue VEGF to cross the blood–brain barrier to promote angiogenesis, which further increases vessel density.^51^

## 3 MECHANISMS OF AEROBIC EXERCISE ON AUTOPHAGY

AMPK maintains and regulates cellular energy metabolism, which is closely related to the occurrence and development of cancer cells.^52^ AMPK acts as a tumor suppressor early in the disease course by inhibiting mammalian target of rapamycin (mTOR), inducing cell autophagy to regulate tumor growth,^53^ mTOR is a central protein that inhibits cell autophagy, and has a negative regulatory effect on the autophagy core gene ULK1.^54^ The AMPK/mTOR pathway is associated with cell autophagy, and when AMP/ATP levels increase, inhibit downstream mTOR protein function by activated AMPK induces autophagy to occur.^55^ AMPK can also promote cell autophagy by activating ULK1 through direct phosphorylation.^56^ Studies showed that promoting cytotoxic autophagy is able to inhibit the growth of breast cancer cells, by activating AMPK/ULK1 signaling axis.^57^ However, this characteristic of AMPK in turn leads to the drug resistance in later stages of tumors.

AMPK has a bidirectional effect on tumor growth, which is related to energy metabolism in the TME.^58^ AMPK is significantly activated under oncogenic stress conditions, promoting glucose metabolism and angiogenesis in tumor cells.^59^ Knockout of AMPK in animal experiments was found to inhibit tumor growth, especially in preneoplastic lesions.^60^ Thus, inhibiting AMPK with effective interventions at early tumor stages may delay tumor development. However, conversely, AMPK activity is low during energy sufficiency. As a negative regulator of the Warburg effect, AMPK inhibits tumor growth by regulating glycolysis and lipogenesis of tumor cells through regulating HIF‐1α, while the loss of AMPK can promote tumorigenesis by regulating lipid metabolism.^61^

**FIGURE 2:**
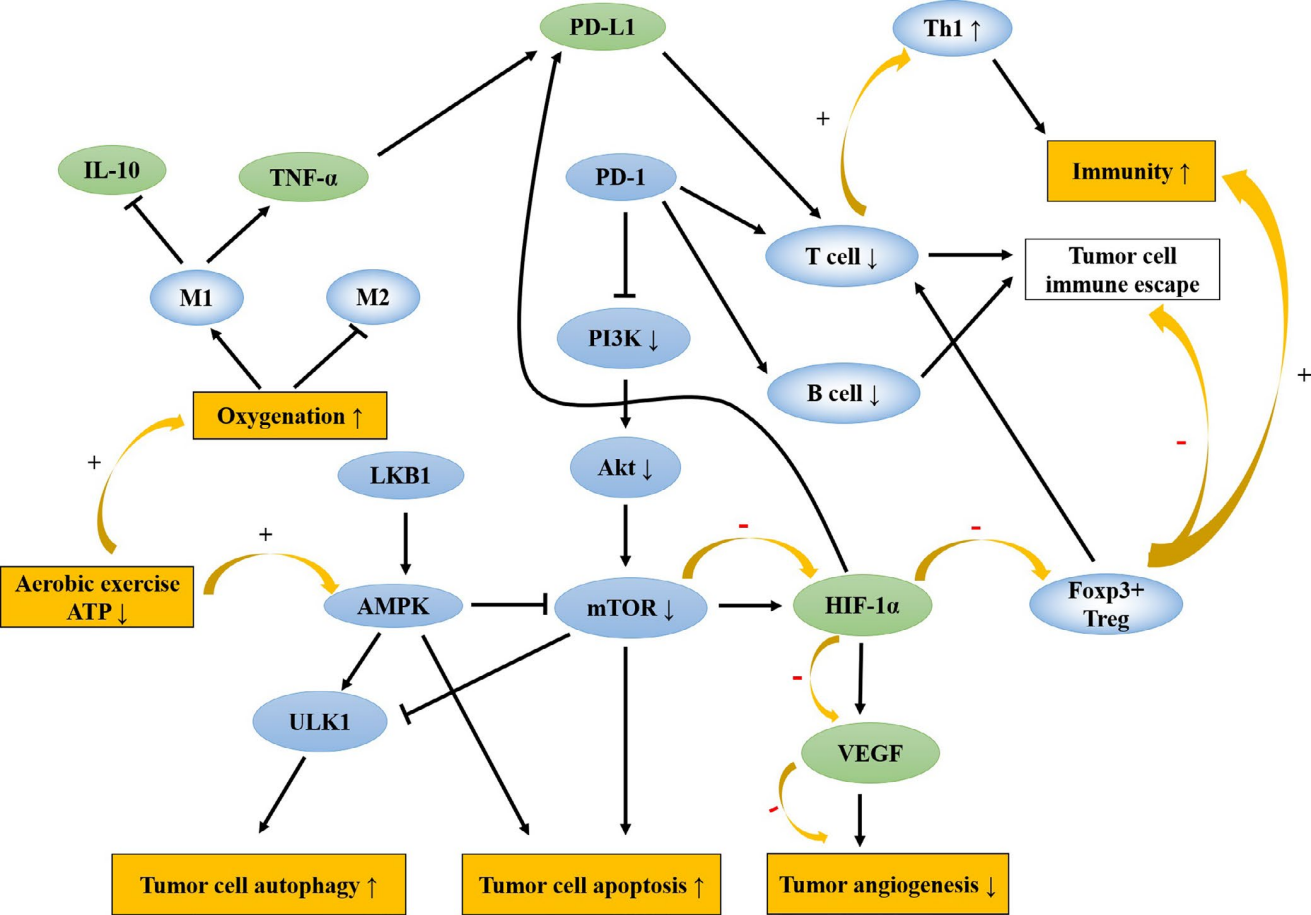
Schematic diagram of the antitumor mechanisms of aerobic exercise. *AMPK*: AMP‐activated protein kinase, *mTOR*: mammalian target of rapamycin, *LKB1*: Liver kinase B1, *CaMKK*‐*β*: Ca^2+^/Calmodulin‐dependent protein kinase kinase, *TNF*‐*α*: tumor necrosis factor‐α, *HIF*‐*1*: hypoxia‐inducible factor‐1, *VEGF*: vascular endothelial growth factor

Exercise is one of the factors that activate AMPK, and low to moderate intensity aerobic exercise can significantly enhance the activity of AMPK.^62^ Liver kinase B1 (LKB1) inhibits mTOR by activating AMPK under low ATP conditions, and the depletion of large amounts of ATP during exercise elevates the AMP/ATP ratio and activates AMPK mediated by LKB1.^63^, ^64^ VEGF is able to promote angiogenesis by modulating cellular metabolism through the activation of the AMPK signaling pathway under conditions of Ca^2+^/calmodulin‐dependent protein kinase kinase (CaMKK‐β) phosphorylation in normal conditions.^65^ While under pathological conditions, stimulation of hypoxia in TME also activates AMPK, which inhibits the expression of multiple angiogenic factors.^66^ Aerobic exercise requires oxygen and constant energy consumption, which activates AMPK to promote angiogenesis in normal cells to exert protective effects through the AMPK/Akt/mTOR signaling pathway^67^ while blocking tumor cell metastasis under pathological conditions. Similar to its role in tumor metastasis, the antiangiogenic effects of AMPK are mostly dependent on the activation of upstream kinase LKB1 and CaMKK‐β.^68^ Considering the characteristic of AMPK, it is recommended that patients undergo aerobic exercise intervention at the initial stage of tumorigenesis such that the cellular autophagic activity of AMPK is exerted to inhibit tumor growth and metastasis (Figure 2).

## 4 MECHANISMS OF AEROBIC EXERCISE ON APOPTOSIS

Tumor cells are characterized by resistance to anoikis, the PI3K/Akt signaling pathway is associated with anoikis resistance.^69^ Insulin and insulin‐like growth factors (IGFs) stimulate nutrient uptake by tumor cells through the PI3K/Akt/mTOR signaling pathway to promote proliferation.^70^ Akt is associated with the anoikis resistance of tumor cells, activated Akt increases the motility of tumor cells.^71^ AMPK, when activated by LKB1, can promote anoikis and thereby inhibit tumor metastasis.^72^ Aerobic exercise decreases circulating levels of insulin, IGF1, and leptin, which is accompanied by altered metabolic signaling in tumor cells, as shown by increased AMPK activation and decreased levels of activated PI3K, Akt, and mTOR,^73^, ^74^ inhibits aerobic glycolysis in cancer cells and induces tumor cell apoptosis (Figure 2).

## 5 MECHANISMS OF AEROBIC EXERCISE ON IMMUNE FUNCTION

### 5.1 Specific immunity

The PD‐1/PD‐L1 signaling pathway is an important component of tumor immunosuppression. T cells are potent immune cells against solid tumors, while PD‐1 inhibits downstream PI3K/Akt signals and induces T‐cell apoptosis.^75^ PD‐L1 causes T‐cell exhaustion and generates immune tolerance, which contributes to tumor immune escape.^76^ Animal experiments showed that the inhibition of CD8^+^T cells causes cancer development.^77^ TME induces the massive generation of regulatory T cells (Treg cells), which inhibit the function of effector T cells.^78^ FoxP3 is an important marker for Treg cells, and the expression of FoxP3^+^Treg is associated with the immune escape of tumor cells, while HIF‐1α can upregulate molecules that attract FoxP3^+^Treg.^79^, ^80^ CD4^+^FoxP3^+^Treg cells are responsible for inducing dominant immune tolerance to tumors,^81^ determined by the ratio between CD8^+^T cells and FoxP3^+^Treg cells.^82^ A relatively high number of FoxP3^+^Treg cells results in a lower ratio, which is significantly associated with shorter overall survival in most solid tumors.^83^ Aerobic exercise is able to increase the effective perfusion of tumor cells, and leads to a faster degradation of HIF‐1α and inhibits FoxP3^+^Treg recruitment.^84^, ^85^ Studies on tumor‐infiltrating B cells have linked cellular immunity and humoral immunity in solid tumors.^86^ Increased antigen presentation by B cells to CD4^+^T cells in the TME, in combination with anti‐PD‐1 therapy, may further enhance immune responses.^87^ However, most studies on immune regulation mediated by tumor‐infiltrating B cells have been performed in vitro, and the mechanism is not well defined. Further studies are therefore needed.

### 5.2 Nonspecific immunity

Activated macrophages can be divided into two types: M1 and M2 macrophages, involved in tumor immune responses.^88^ Inflammatory cytokines released by macrophages can promote the proliferation of tumor cells.^89^ Recent studies showed that tumor necrosis factor‐α (TNF‐α) secreted by macrophages and the inflammatory cytokine interleukin‐1β (IL‐1β) induce the upregulated expression of PD‐L1 via NF‐kB and STAT3 signaling pathways in tumor cells.^90^ Interleukin‐6 (IL‐6) is an immune inflammatory cytokine, and the effects on the inflammatory response depend on the context. IL‐6 enhances inflammatory immune function in normal conditions, while inhibiting the expression of TNF‐α under hypoxia, leading to anti‐inflammatory effects.^91^ In pathological settings, IL‐6 induces macrophage polarization from M1 to M2, causing tumor cell metastasis. M2 promotes proliferation by regulating PD‐L1 expression.^92^ Due to the lactate‐sensitive property, macrophages are activated into M2 in lactate‐rich TME.^93^ Aerobic exercise promotes activation of the M1, improving oxygenation to reduce the exposure to lactate, thereby enhancing M1 activation or decreasing the activation of M2.^94^ Moreover, epinephrine surges during aerobic exercise, mobilizing NK cells into the circulation to inhibit tumor growth.^95^, ^96^ However, with new classification of different M2 subgroups,^97^ whether aerobic exercise will continue this shift is worth investigating in the future (Figure 2).

## 6 CONCLUSION

Aerobic exercise acts as a tumor suppressor with regard to processes occurring in the hypoxic tumor microenvironment, cell autophagy versus apoptosis, and body immune function through regulation of the corresponding mechanisms and triggering certain cytokine signaling pathways. Some new ideas deserve continued exploration: determining whether newly discovered subgroups of cytokines influence the positive effects of aerobic exercise on tumors; adjusting corresponding exercise prescriptions based on the bidirectional effects of certain cytokines at different lesion stages; identifying the potential effects of exercise time and intensity on tumor growth. The study of the link between aerobic exercise and tumors still needs further exploration in the future, so as to develop more effective clinical treatment regimens.

## CONFLICT OF INTEREST

No conflict of interest in this study.

## AUTHOR CONTRIBUTIONS

NJ and MD planned the structure of the manuscript. NJ wrote the manuscript and designed the figures. YZ and XD participated in its design and coordination, and helped to draft the manuscript. All authors read and approved the final manuscript.

## ETHICAL STATEMENT

This is a review article and the need for ethics approval and consent was waived.

## Data Availability

This is a review article. Data sharing not applicable to this article as no datasets were generated or analyzed during the current study. All the figures in the manuscript are original.
